# Glass Microbubble Encapsulation for Improving the Lifetime of a Ferrofluid-Based Magnetometer

**DOI:** 10.3390/mi16050519

**Published:** 2025-04-28

**Authors:** Chenchen Zhang, Srinivas Tadigadapa

**Affiliations:** 1 Materials Research Institute, Department of Electrical Engineering, Penn State University, University Park, PA 16802, USA; chenchen_zhang@senodia.com; 2 Department of Electrical and Computer Engineering, Northeastern University, Boston, MA 02115, USA

**Keywords:** glass microbubble, chip-scale packaging, quartz resonator, Metglas, magnetometer

## Abstract

In this paper, we explore the use of chip-scale blown glass microbubble structures for MEMS packaging applications. Specifically, we demonstrate the efficacy of this method of packaging for the improvement of the lifetime of a ferrofluid-based magnetoviscous magnetometer. We have previously reported on the novel concept of a ferrofluid based magnetometer in which the viscoelastic response of a ferrofluid interfacial layer on a high frequency shear wave quartz resonator is sensitively monitored as a function of applied magnetic field. The quantification of the magnetic field is accomplished by monitoring the at-resonance admittance characteristics of the ferrofluid-loaded resonator. While the proof-of-concept measurements of the device have been successfully made, under open conditions, the evaporation of the carrier fluid of the ferrofluid continuously changes its viscoelastic properties and compromises the longevity of the magnetometer. To prevent the evaporation of the ferrofluid, here, we seal the ferrofluid on top of the micromachined quartz resonator within a blown glass hemispherical microbubble attached to it using epoxy. The magnetometer design used a bowtie-shaped thin film Metglas (Fe_85_B_5_Si_10_) magnetic flux concentrator on the resonator chip. A four-times smaller noise equivalent, a magnetic field of 600 nT/√Hz at 0.5 Hz was obtained for the magnetometer using the Metglas flux concentrator. The ferrofluid-based magnetometer is capable of sensing magnetic fields up to a modulation frequency of 40 Hz. Compared with the unsealed ferrofluid device, the lifetime of the glass microbubble integrated chip packaged device improved significantly from only a few hours to over 50 days and continued.

## 1. Introduction

**Figure 1:**
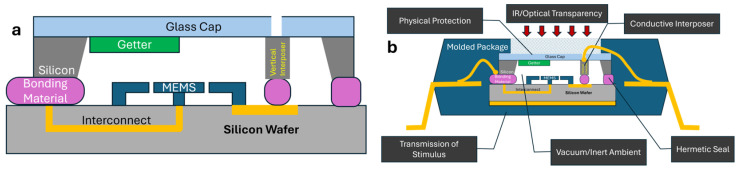
Schematic illustration of typical hierarchical MEMS packaging. (**a**) The die level cap wafer is used to provide the necessary hermetic seal through the bonding material. Getters can be used to achieve and maintain vacuum within the package, and interposers provide electrical feedthroughs for connection to the final package. (**b**) Plastic molding is used to secure the die in place with electrical connections to pins and integration of any necessary optical windows.

MEMS packaging has been a topic of investigation for the last couple of decades addressing the needs to both protect the mechanically fragile micromachined structures from physical damage, as well as to provide specific operational ambience for optimal performance [1,2,3,4]. Typically, such packaging has been achieved through a hierarchical sequence through the integration of a wafer-level cap to provide necessary ambient for operation, hermetic seals as needed, and electrical feedthroughs [5,6,7,8] and a die level molding providing the final encapsulation to accomplish the necessary mechanical enclosure and electrical pin outs for handling, mounting, and integration onto circuit boards. Figure 1a shows a schematic illustration of the encapsulation that is typically accomplished using wafer-level bonding of the cap wafer, and Figure 1b shows the capped wafer embedded within a molded package. Figure 1b also lists the various functions the packaging cap needs to accomplish.

Wafer-level packaging is accomplished through the use of a cap wafer, which typically consists of either a silicon, an oxidized silicon, or a silicon-on-insulator wafer and occasionally specialized wafers such as zinc selenide, calcium fluoride, etc., depending upon the application. In most cases, the silicon handle layer is bulk etched to create the necessary cavity within which the MEMS structures are located. Furthermore, thin layers of sputter-deposited metals such as titanium, zirconium, vanadium and iron alloys or screen-printed commercial getters are used to adsorb any outgassing products within the package cavity. These getter layers are thermally activated either during the cap sealing step or separately following the cap sealing. The cap wafers are attached to the MEMS substrate wafer through various bonding processes. These include direct wafer bonding [9], anodic bonding [10], eutectic bonding [11], solder bonding [12], glass-frit bonding [13], and epoxy bonding [14]. Except for direct wafer and anodic bonding, all other bonding processes use an intermediate layer to accomplish the hermetic seal of the cap. Any surface topography or non-uniformity due to lead transfer patterns or other imperfections lend both direct wafer and anodic bonding ineffective for forming hermetic seals. Furthermore, the annealing processes for direct wafer bonding can be high and often not suitable for MEMS packaging processes. Therefore, in most cases, the cap wafers are sealed using intermediate layers listed above. Anodic, glass-frit, and eutectic bonding involve a temperature of ~400 °C, whereas solder and adhesive bonding could be performed at a temperatures of ≤200 °C. Given the very small volume of chip level MEMS packages, most of the vacuum degradation occurs primarily due to outgassing from the surfaces within the cavity. Getters are able to absorb and trap most of the outgassing species, such as O_2_, H_2_, OH, CO, CO_2_, other small organic molecules, etc. However, they are not effective at trapping inert gas molecules such as Ar and He.

**Figure 2:**
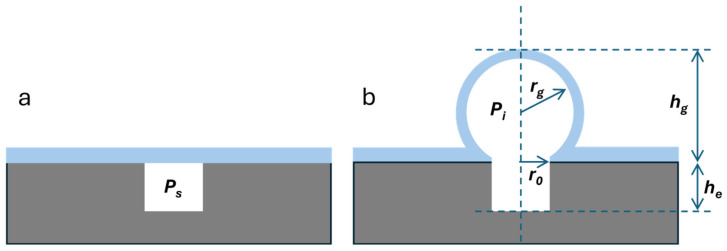
Schematic illustration showing (**a**) the bonding of borosilicate glass (blue) to a silicon (gray) wafer with an etched cavity at a pressure of Ps and a temperature of Ts and (**b**) after the glass blowing process at a temperature of Tf. The various geometric parameters of the structure are also shown.

Here, we describe a novel capping process that can be used for MEMS and sensor packaging and can provide a robust encapsulation for various applications. The process involves chip-scale blowing of borosilicate glass [15] to realize the package cavity. The blown glass structure can be formed directly over the MEMS structure following the anodic bonding of the borosilicate glass to the MEMS substrate, or it can be attached using any of the intermediate layers discussed above. The on-chip glass blowing process results in the creation of a vacuum within the cavity, the value of which is dependent upon both the pressure and temperature at which the initial sealing is done, as well as the final volume of the microspherical shell and the temperature at which the glass blowing process is performed. Figure 2 schematically illustrates the process of glass blowing and the various geometric parameters. The pressure $P_{i}$ inside the blown structure can be given by the expression [16]:

(1) $$P_{i}=\frac{P_{s}\frac{T_{f}}{T_{s}}}{1+\frac{h_{g}}{6r_{0}^{2}h_{e}}\left(h_{g}^{2}+3r_{0}^{2}\right)}$$

where $P_{s}$ is the pressure at which the cavity was sealed in Figure 2a, $T_{f}$ is the temperature at which the glass is blown and $T_{s}$ is the temperature at which the cavity is sealed, $r_{0}$ is the radius and $h_{g}$ is the depth of the etched circular cavity in silicon, and $h_{e}$ is the height of the blown spherical shell, as shown in Figure 2b. For $P_{s}$ of 100 Torr, $T_{f}$ of 900 °C, $T_{s}$ of 400 °C, $h_{g}$ of 1 mm, $r_{0}$ of 0.125 mm, $h_{e}$ of 0.25 mm, the $P_{i}$ is calculated to be 14 Torr. Use of the getter during the glass blowing process is expected to reduce the pressure within the cavity even further. The glass blowing process is much simpler than elaborate cap wafer preparation processes and provides a mechanically strong enclosure for MEMS devices, as we will show in the rest of this paper.

Easy-to-use, low-cost, chip-scale, ultra-sensitive magnetometers are attractive for several applications, such as position sensors, orientation sensors, and biomedical imaging and diagnosis [17]. Many innovative approaches have been proposed and investigated to explore sensitive magnetometers. Superconducting quantum interference devices (SQUIDs) are the most sensitive magnetic field sensor, which are reported with <4.5 fT/√Hz resolution [18]. Atomic magnetometers have also shown comparable sensitivity [19,20]. However, SQUIDs require cryogenic cooling to operate, whereas the sensor vapor cell in atomic magnetometers must be heated to temperatures >150 °C. Other magnetic field sensors such as fluxgate sensors [21], giant magnetoresistance (GMR) spin valve [22], magnetoelectric sensors [23], and magnetoflexoelastic resonator-based sensors [24] have been demonstrated with sensitivities ranging from 10 nT to 100 pT. Our group has demonstrated a novel concept for magnetic sensing that is based upon a magnetoviscoelastic effect in ferrofluids [25]. Ferrofluids are emulsions of nanometer-sized ferroparticles suspended in a carrier liquid. The ferromagnetic particles are able to organize and aggregate spontaneously under the influence of external magnetic fields, resulting in large viscoelastic property changes in the ferrofluids, and this phenomenon is known as the magnetoviscous effect [26,27]. Our previous work proposed the idea of a novel magnetometer, in which a ferrofluid was loaded atop a high-frequency quartz shear wave resonator, and the induced changes in the viscoelastic properties of the ferrofluids due to applied magnetic fields are sensed by monitoring the changes the at-resonance impedance characteristics of the quartz resonator. The proof of concept work demonstrated a very promising magnetic sensitivity of 1.5 nT/√Hz [25].

However, the lifetime of the device was limited only a few hours, since, without any sealing of the ferrofluids liquid, the base fluid continuously evaporated and dried out. In this paper, we will specifically address this issue and demonstrate the application of a microfabricated blown glass microbubble structure to achieve chip-scale device packaging that allows the ferrofluids to be hermetically sealed atop the resonator to achieve a magnetometer with robust lifetime.

## 2. Fabrication and Experimental Setup

### 2.1. Quartz Resonator Chip

**Figure 3:**
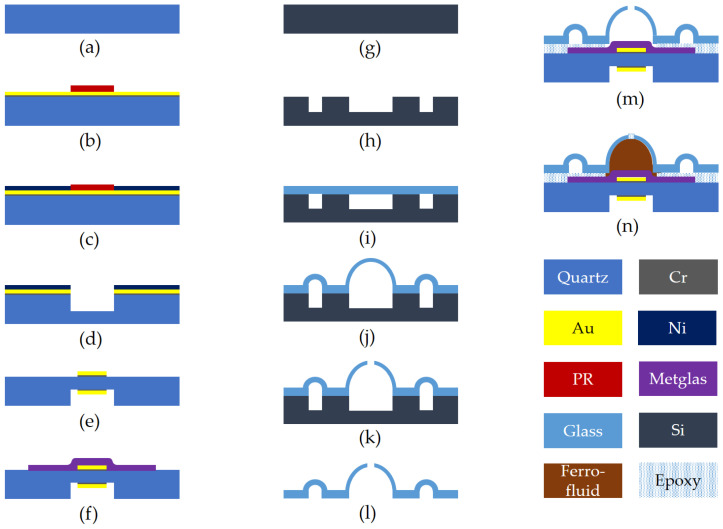
Includes cross-sectional schematics of a step-by-step process flow chart to systematically present the key fabrication stages. (**a**) quartz wafer, (**b**) Cr/Au deposited and photoresist is patterned, (**c**) nickel electroplating, (**d**) photoresist is removed and quartz etched in ICP-RIE, (**e**) Cr/Au electrode is patterned on otherside, (**f**) Metglas^®^ film is deposited on the front side of the wafer and patterned into the bowtie shape, (**g**) silicon wafer, (**h**) Patterned and DRIE to create cavities, (**i**) borosilicate glass 100 µm thick is anodically bonded to silicon at 1.35 atm, (**j**) glass is blown by rapidly heating to above the softening temperature, (**k**) hole is drilled on top of the glass bubble, (**l**) the handle silicon wafer is etched in KOH solution, (**m**) glass bubble wafer is carefully aligned to the quartz resonator wafer and bonded using epoxy, and (**n**) the glass bubble is filled with ferrofluid using a syringe and sealed with Devcon^®^ expoxy on top to complete the device.

**Figure 4:**
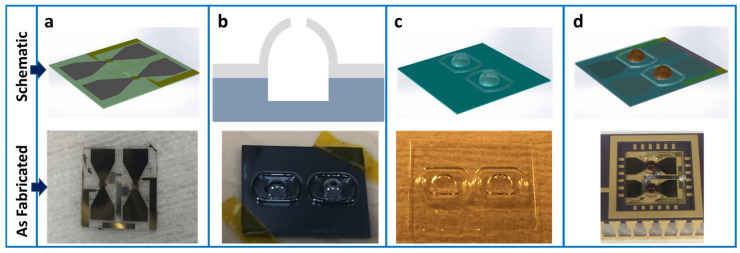
Delineates the sequential fabrication steps of the chip-scale sealed magnetoviscous magnetometer. (**a**) Illustrates the fabrication process of the quartz resonator chip. (**b**) Showcases the fabrication of the glass microbubble chip. (**c**) The silicon substrate undergoes wet etching, a process that removes material to reveal the delicate glass microbubble structure. (**d**) Depicts the two resonator and glass microbubble structures and packaging of the ferrofluid magnetometer.

The fabrication process of the micromachined quartz resonators commenced with the cleaning of a 100 μm thick, 1-inch diameter polished AT-cut quartz crystal substrate in Nanostrip™ (Santa Ana, CA, USA) solution for a duration of 30 min, as shown in Figure 3a. Subsequently, layers of chromium and gold (Cr/Au) with thicknesses of 15 nm and 150 nm, respectively, were deposited onto one surface of the quartz substrate via evaporation. The Cr/Au layer serves as a seed layer for further processing. The substrate was then coated with SPR-220 photoresist, which was spin-coated to a thickness of 15 μm, as shown in Figure 3b, to define two resonator regions, each with a diameter of 1 mm. A 10 μm layer of nickel was electroplated onto the patterned areas to function as a hard mask for the ensuing etching procedure, as shown in Figure 3c. Following the removal of the photoresist, as shown in Figure 3d, the designated resonator regions were thinned to a final thickness ranging between 8 and 15 μm, employing a high-speed plasma etching technique typically used for silicon dioxide [28]. After the residual nickel mask was thoroughly stripped away, layers of chromium (20 nm) and gold (100 nm) were deposited through evaporation. These layers were then lithographically patterned and subjected to wet etching to create the bottom electrode. The quartz substrate was subsequently flipped, and a precisely aligned lithography process was performed to define the top electrode. This was achieved by lifting off evaporated films of chromium (20 nm) and gold (100 nm), as shown in Figure 3e. Both the top and bottom electrode patterns were extended to one edge of the chip to facilitate wire bonding after the integration of a glass microbubble above the resonator. To complete the fabrication, a 500 nm thick layer of Metglas^®^ (Conway, SC, USA), known for its high relative permeability, was sputtered and patterned into a bowtie shape. This structure acts as a magnetic flux concentrator and is located on the flat, unetched side of the resonator, as shown in Figure 3f. Figure 4a shows a schematic representation and an optical image of the completed micro-quartz crystal resonator (μQCR) with two resonators on the chip and each chip measuring 9 mm × 9 mm in size.

### 2.2. Glass Microbubble Chip

A silicon wafer with a thickness of 550 μm and a diameter of 4 inches, as shown in Figure 3g, was patterned and subjected to deep reactive ion etching (DRIE) to create elongated circular pits with a depth of 250 μm, as shown in Figure 3h. In this work, a Borofloat^®^ (New York, USA) 33 glass wafer, 100 μm thick and 4 inches in diameter, was anodically bonded to the etched silicon wafer, as shown in Figure 3i. This bonding process was conducted under a nitrogen atmosphere pressurized to 1.35 atm. The pressurized gas was hermetically sealed within the circular cavities formed between the glass and silicon wafers. The bonded wafer was then diced into individual dies measuring 8 mm × 9 mm. Each die was placed in a rapid thermal annealing (RTA) chamber and rapidly heated to 850 °C for 3 min. At this elevated temperature, the borosilicate glass softens, and the trapped nitrogen within the silicon cavities expands, causing the softened borosilicate glass to deform into semi-spherical microbubbles, as depicted in Figure 3j and schematically and optically captured in Figure 4b.

For chip-scale glass blowing, the bonded glass has to meet two criteria. The first is that it is compatible with anodic bonding to silicon at temperatures below 500 °C and well below the softening point of the glass, and second the softening point of the glass, at which its viscosity decreases rapidly, needs to be below 1000 °C. Anodic bonding allows for a hermetic seal at the interface. Several borosilicate glass wafers can be used for anodic bonding to silicon at around 400 °C. In the anodic bonding process, the anode repels sodium ions and attracts oxygen ions towards the Si-SiO_x_ interface to create a Si-O-Si interface region, which has been found to be mechanically stronger than either silicon or glass [29]. Glasses for anodic bonding to silicon include Corning^®^ Pyrex 7740, 7070, and 9626 (Corning, NY, USA); Hoya^®^ SD2 (Tokyo, Japan); or Schott^®^ Borofloat (New York, USA), all borosilicate formulations, each with different sodium contents (2–5%) [30,31]. Lithium-based glasses have also been explored for anodic bonding at an even lower temperature of 250 °C [32]. Furthermore, all these glasses soften between 800 and 1100 °C. This is a critical need, as it is difficult to accurately control the temperature–time of glass blowing once it reaches very high values. Below 1000 °C, we have been able to obtain sufficient control of the glass blowing process. Finally, once the glass blowing process is complete, the chips need to be cooled very rapidly to below the softening temperature to prevent collapse of the bubble, and at temperatures below 1000 °C, the kinetics can be well controlled without the use of forced convective cooling. This is one reason why chip-scale glass blowing with pure silica (SiO_2_) wafers exhibiting a softening point at >1800 °C is very difficult and needs specialized equipment that has not been used here.

The fabricated microbubbles measured 1.6 mm with a tolerance of ±0.2 mm in diameter. The detailed microfabrication reliability and comparison accuracy between measurement data and theoretical prediction, as modeled in Equation (1), could be found in a previous publication [15]. Alongside the central microbubble, a surrounding ring-shaped glass structure was also expanded, forming a moat-like barrier to prevent adhesive overflow onto the central region during subsequent sealing processes. Following the thermal shaping of the microbubbles, a 200 μm diameter hole was drilled on top of each microbubble using a diamond-tipped micro-drill, as shown in Figure 3k, allowing for injection of the ferrofluid later. Finally, the silicon substrate was entirely dissolved using a 22% (wt.) potassium hydroxide wet etchant at 80 °C over a period of 30 h, leaving behind the delicate glass microbubble chip, as illustrated in Figure 3l and as completed in Figure 4c.

### 2.3. Ferrofluid Magnetometer Packaging

The quartz resonator chip was securely mounted within a standard dual in-line ceramic package using silver epoxy, which simultaneously established an electrical connection to the bottom electrode. The flat surfaces of the glass microbubble chip were coated with a thin layer of Devcon^®^ (Ohio, USA) 10-min epoxy. The two chips, the resonator and the microbubble, were then precisely aligned, brought into contact, and gently pressed together, as shown in Figure 3m. The moat-like structure surrounding the central microbubble served as a reservoir to capture any excess Devcon^®^ epoxy that might escape during the bonding process, thereby preventing it from contaminating the central resonator region. Once the Devcon^®^ epoxy had fully cured, approximately 1 µL of EMG 911 ferrofluid was carefully injected into the microbubble through the top opening using a BD Ultra-Fine^®^ (Pennsylvania, USA) 31-gauge syringe. The opening was subsequently sealed with a small piece of Kapton^®^ (Delaware, USA) tape and an additional layer of Devcon^®^ epoxy, which was allowed to cure for 24 h. Figure 3n shows the cross-sectional schematic of the final integrated device architecture. Figure 4d provides a 3D schematic view of the device and a photograph of the fully assembled device, showcasing the ferrofluid encapsulated within the glass microbubble chip positioned above the quartz resonator region, all housed within the dual-in-line package.

### 2.4. Experimental Setup

**Figure 5:**
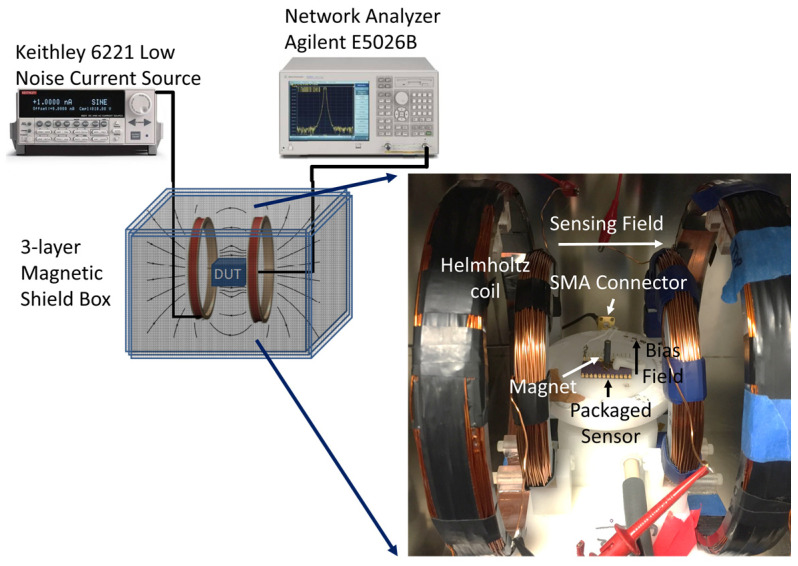
The experimental setup, where a low-noise current source is utilized to drive Helmholtz coils, generating a modulated magnetic field within the magnetically shielded box. The device under test (DUT) is connected to a network analyzer for impedance measurements. The inset image provides a detailed view of the setup inside the shielded enclosure: the packaged device is mounted on a stage at the center of the Helmholtz coils and linked to the network analyzer via an SMA connector. This configuration ensures precise control and measurement of the magnetic field’s influence on the device.

The assembled device was positioned at the center of a Helmholtz coil and interfaced with a network analyzer via a high-frequency SMA connector. The entire experimental setup was enclosed within a three-layer mu-metal box to ensure magnetic shielding. To apply a constant out-of-plane bias magnetic field (perpendicular to the resonator’s surface), a permanent magnet was placed approximately 3 mm beneath the ceramic package, near the resonator/ferrofluid interface. The resonance characteristics of the micromachined quartz resonator were measured using an Agilent E 5061B (Colorado, USA) network analyzer, which is capable of capturing 1601 impedance measurements across the specified frequency range. Figure 5 illustrates the experimental setup employed for magnetic field modulation and data acquisition, with an inset image providing a closer view of the Helmholtz coil and the packaged device inside the shielded enclosure.

## 3. Results and Discussion

### 3.1. Characterization of Quartz Resonator

**Figure 6:**
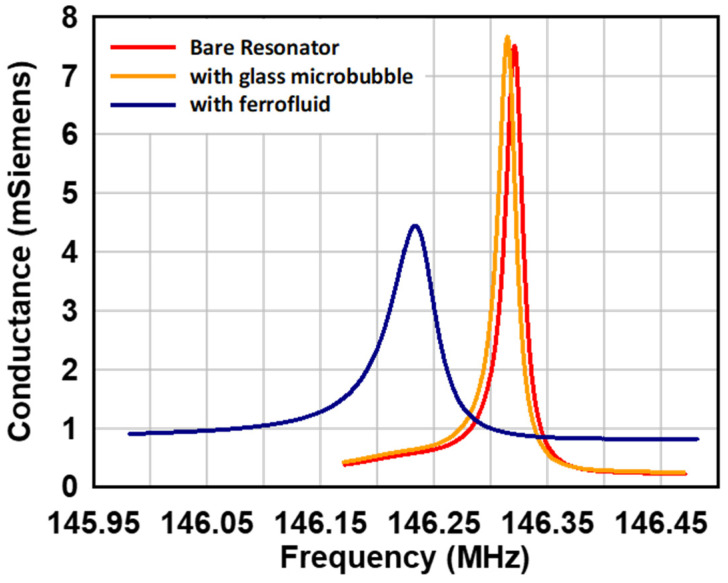
Resonance characteristics after each packaging step.

The resonance performance of the fabricated quartz resonator was evaluated at each stage of the packaging process. Initially, the quartz resonator, featuring only the top and bottom electrodes without the Metglas^®^ flux concentrator, was wire-bonded into a ceramic dual inline package. The package was then connected to a network analyzer via SMA connections. The conductance as a function of frequency, plotted in red in Figure 6, revealed a resonance frequency of 146.3209 MHz, indicating that the quartz resonator had been etched to a thickness of 11.4 µm. The quality factor of the resonator was calculated to be 7726, reflecting its high performance. Subsequently, the resonator was reevaluated 6 h after the glass microbubble was attached to the quartz resonator, ensuring that the packaging process did not compromise its resonance characteristics. Although Devcon^®^ epoxy is designed to cure within 15 min, the additional curing time ensured the complete removal of solvent vapors from the enclosed cavity above the resonator. The conductance measured after this process is represented by the orange curve in Figure 6. A slight leftward shift in the resonance frequency was observed, indicating the addition of a small mass on the resonator surface due to the adsorption of epoxy solvent vapors trapped within the glass microbubble enclosure during curing. Despite this shift, the quality factor of the resonator remained nearly unchanged after the microbubble chip was attached. Finally, the resonance characteristics were measured after the ferrofluid was loaded and sealed within the microbubble cavity. A small neodymium magnet was used to induce the formation of a viscoelastic ferrofluid interfacial layer on the quartz resonator surface. The blue curve in Figure 6 illustrates the resonance characteristics after ferrofluid loading compared to the original resonance profile. The new resonance frequency was determined to be 146.2337 MHz, reflecting a decrease of 87.1825 kHz due to the viscoelastic loading effect of the ferrofluid. Additionally, the quality factor of the resonator decreased to 2753 at the new resonance frequency. Table 1 summarizes the resonance characteristics of the three fabricated quartz resonators in this study. Among the three devices, Device 1, although it has the Metglas^®^ flux concentrator (Conway, SC, USA), has a very poor *Q*-factor and did not show good magnetometer performance. Device 2 did not have the flux concentrator structure and was primarily used to develop the integration of the bubble structure to the resonator. Device 3 has the integrated Metglas^®^ flux concentrator structure and exhibited an excellent *Q*-factor, as well as the best response to magnetic fields amongst the three devices studied here.

### 3.2. Response to Magnetic Field

**Figure 7:**
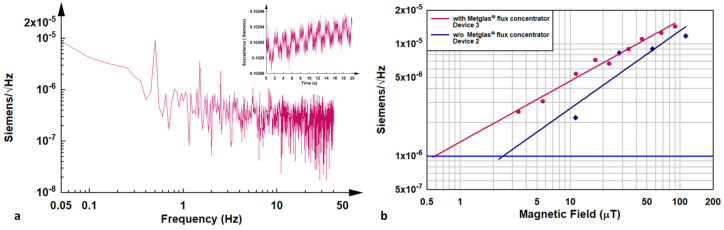
The analysis of the measured susceptance response spectrum. (**a**) A fast Fourier transform (FFT) algorithm is applied to the time domain data, converting it into the frequency domain. The inset image within Figure 4a displays the real-time susceptance response of the resonator to a modulated magnetic field, demonstrating its dynamic behavior. (**b**) Plots of the amplitude of the FFT peak signal at the modulation frequency (0.5 Hz) as a function of the applied modulation magnetic field. This plot highlights the relationship between the magnetic field strength and the resonator’s response, providing insights into the device’s sensitivity and performance under varying magnetic conditions.

A low-frequency sensing magnetic field was applied using Helmholtz coils, which perturbed the self-assembled and ordered interfacial viscoelastic ferrofluid layer. The resulting magnetoviscoelastic changes in this layer were monitored by tracking the at-resonance susceptance of the resonator. The inset image in Figure 7a illustrates the real-time response of Device 3 to a sinusoidal magnetic field with a peak value of 33.6 µT. The time domain susceptance response data were converted into the frequency domain using a fast Fourier transform (FFT), as shown in Figure 7a. Figure 7b displays the amplitude of the FFT peak signals at the modulation frequency (0.5 Hz) as a function of the applied magnetic field for Device 2 and Device 3. The noise level was determined by the FFT signal at 0.5 Hz in the absence of an applied magnetic field. This analysis demonstrates the sensitivity of the resonator to magnetic field variations and its ability to detect magnetoviscoelastic changes in the ferrofluid layer. From the analysis, it is evident that the predicted minimum detectable sensitivity of the glass microbubble-packaged ferrofluid µQCR is 600 nT for Device 3 and 2.5 µT for Device 2. The sensitivities obtained from the three fabricated devices are summarized in Table 2, highlighting the variation in performance across the devices and the potential for optimizing the design to achieve higher sensitivity.

### 3.3. Lifetime of Packaged Device

The concept of utilizing ferrofluid loaded onto a high-frequency quartz shear wave resonator and monitoring the at-resonance impedance changes in response to external magnetic fields for magnetic field sensing was previously demonstrated in [9]. However, the focus of this work was to advance the technology by developing a chip-scale packaging method to enhance the long-term reproducibility and operational lifetime of the magnetoviscous magnetometer. In the earlier open version of the device, the lifetime was limited to just a few hours due to the evaporation of the ferrofluid solvent, which led to continuous drift and eventual sensor failure.

**Figure 8:**
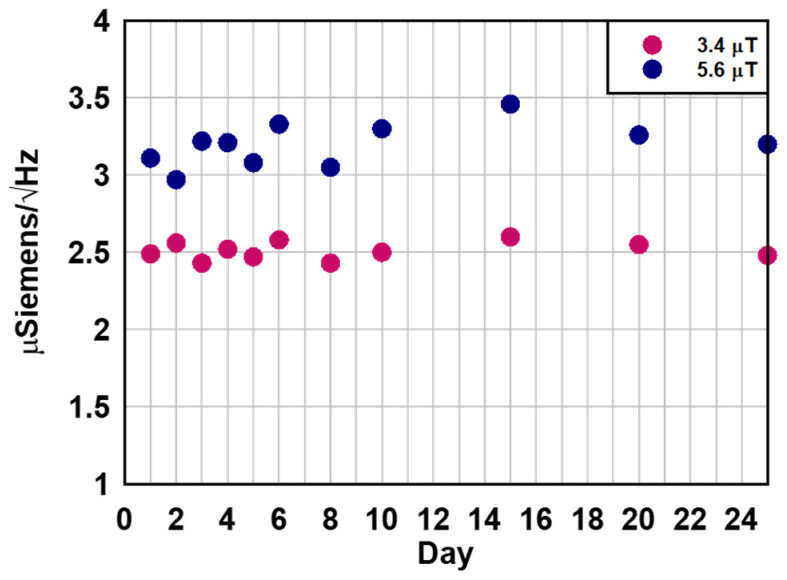
Frequency shift of Device 3 under two applied external magnetic field as a function of time.

To address this limitation, this work introduced a glass microbubble-based packaging strategy, which provides hermetic sealing of the ferrofluid atop the resonator. This approach significantly improves the sensor’s lifetime and stability. To demonstrate the effectiveness of this packaging method, the sensitivity of two resonators (Device 2 and Device 3) was monitored over time. Both devices have maintained reproducible magnetic sensitivity for over 25 days and continue to function reliably. Device 1 has shown reproducible frequency shifts under external magnetic fields for more than 50 days. Figure 8 illustrates the repeatable sensitivity performance of Device 3 under two externally applied magnetic fields over several days, highlighting a substantial improvement over the original design’s few hours of operation.

## 4. Conclusions

In summary, this work has successfully demonstrated a chip-scale packaging approach using blown glass microbubble cavities, which can be applied to a wide range of MEMS and microscale sensors. The hermetically sealed cavities enable the creation of controlled environments, such as vacuum or specific fluid ambient, around microelectromechanical sensor structures. This chip-scale packaging method not only ensures a compact form factor but also facilitates wafer-level integration, paving the way for scalable and robust sensor applications.

## Figures and Tables

**Table 1:** Resonance characteristics of three fabricated quartz resonators.

Device	*f*_0_ *	*Q* *	*Q*′ *	*t* *	Metglas Patterns	Bias Field *
1	131.0	1500	440	12.7	Yes	5
2	146.3	7726	2753	11.4	No	10
3	122.6	NA	10779	13.6	Yes	3

* *f*_0_: resonance frequency in MHz, *Q*: quality factor of quartz resonator before ferrofluid loading, *Q*′: quality factor of quartz resonator after ferrofluid loading, and *t*: thickness of the resonator in µm. Bias field: in mT.

**Table 2:** Summary of obtained sensitivity from three devices.

Device Number	Minimum Measured Magnetic Field (µT)	Minimum Predicted Magnetic Field (µT)	Modulation Frequency (Hz)
1	200	15	0.01
2	11.2	2.4	0.5
3	3.4	0.6	0.5

## Data Availability

The original contributions presented in the study are included in the article, further inquiries can be directed to the corresponding author.
